# Natural Photosensitizers in Antimicrobial Photodynamic Therapy

**DOI:** 10.3390/biomedicines9060584

**Published:** 2021-05-21

**Authors:** Ece Polat, Kyungsu Kang

**Affiliations:** 1 Natural Product Informatics Research Center, Korea Institute of Science and Technology, Gangneung 25451, Gangwon-do, Korea; ecepolat@yahoo.com; 2 Division of Bio-Medical Science Technology, KIST School, University of Science and Technology (UST), Gangneung 25451, Gangwon-do, Korea

**Keywords:** antimicrobial photodynamic therapy, natural photosensitizers, natural extracts, antibiotic resistance, model organisms, biophotonics, light, curcumin

## Abstract

Health problems and reduced treatment effectiveness due to antimicrobial resistance have become important global problems and are important factors that negatively affect life expectancy. Antimicrobial photodynamic therapy (APDT) is constantly evolving and can minimize this antimicrobial resistance problem. Reactive oxygen species produced when nontoxic photosensitizers are exposed to light are the main functional components of APDT responsible for microbial destruction; therefore, APDT has a broad spectrum of target pathogens, such as bacteria, fungi, and viruses. Various photosensitizers, including natural extracts, compounds, and their synthetic derivatives, are being investigated. The main limitations, such as weak antimicrobial activity against Gram-negative bacteria, solubility, specificity, and cost, encourage the exploration of new photosensitizer candidates. Many additional methods, such as cell surface engineering, cotreatment with membrane-damaging agents, nanotechnology, computational simulation, and sonodynamic therapy, are also being investigated to develop novel APDT methods with improved properties. In this review, we summarize APDT research, focusing on natural photosensitizers used in in vitro and in vivo experimental models. In addition, we describe the limitations observed for natural photosensitizers and the methods developed to counter those limitations with emerging technologies.

## 1. Introduction

After penicillin was identified as a product of *Penicillium notatum* by Alexander Fleming in 1928, its widespread consumption was noted in the early 1940s. In 1944, 50% of the clinical isolates of *Staphylococci* sp. were unexpectedly shown to exhibit resistance to penicillin [1]. Extensive antibiotic (mis)use has led to the spread of more resistant bacteria to the environment, and antimicrobial resistance (AMR) is an increasing threat to humans. By 2050, 10 million deaths per year are expected to be related to AMR [2]. Antibiotic-resistant pathogens are emerging threats to human life and are classified as methicillin-resistant *Staphylococcus aureus* (MRSA), vancomycin-resistant *Enterococcus faecalis*, multidrug-resistant mycobacteria, Gram-negative pathogens, and fungi [1].

Antibiotic resistance and antibiotic pollution are common problems worldwide. Antibiotic resistance in infections potentially results in sepsis and even ineluctable systemic inflammation and organ failure [3]. Medicine is not the only source of antibiotic resistance. Aquaculture production, livestock, and pets consume and waste a high rate of antibiotics. Some probiotics and plant extracts containing essential oils have been introduced as alternative treatments to overcome excess antibiotic usage in aquaculture [4]. Notably, every use of antibiotics can create selective pressure for mutation and the development of drug resistance [5]. Overuse or misuse of antibiotics can increase the rate of AMR. Some country-based strategies have been implemented to overcome AMR. Additionally, in 2014, the World Health Organization (WHO) published a global report on AMR [6].

Antimicrobial photodynamic therapy (APDT) is a challenging method to overcome excess antibiotic consumption and limit antibiotic resistance gene transfer. Quorum sensing, vaccines, lectin inhibition, and iron chelation have been used as treatments for drug-resistant microorganisms, and APDT might be considered a favorable technique among them. The photodynamic effect was first described by Oscar Raab in 1904 and first successfully used to treat cancer cells in 1905 [7]. By the time PDT focused on cancer cell treatment, APDT was focused on overcoming antibiotic resistance by targeting bacteria, algae, yeasts, and viruses [8].

In addition to combating AMR and excess antibiotic consumption, APDT can provide strong antibiotic potency. APDT inhibits a broad spectrum of pathogens because its antimicrobial activity originates from reactive oxygen species (ROS) production induced by a unique photochemical reaction. Viera et al. [9] studied APDT efficiency against a broad range of organisms, including bacteria, fungi, and viruses, and reported that APDT is effective against a wide range of organisms. Tissue specificity is another advantage of APDT. Generally, no toxicity is observed in nonphotosensitizer-treated cells or in cells that are not exposed to light. Photosensitizers (PSs) are known to be taken up predominantly by target cells rather than nontarget cells. Only the infected tissue is irradiated, and the PS in unirradiated locations is pharmacodynamically passive. In addition, Park et al. [10] reported that APDT can be used effectively without damaging resident flora or human tissue.

## 2. Key Factors in Antimicrobial Photodynamic Therapy (APDT)

Light, oxygen, and PSs in precise cooperation are the key factors determining APDT efficiency and are responsible for ROS production and the inactivation of the targeted cells [11].

### 2.1. Light Sources

Photobiomodulation has been applied to relieve pain, decrease inflammation, and stimulate the healing of living tissues [12]. Light sources significantly affect PDT. Sunlight with diverse wavelengths activates the PS and causes shallow tissue penetration, thermal effects, and difficulties in controlling the dose [13]. Xenon lamps, light emitting diodes (LEDs), laser beams, and fiber optic devices are alternatives to sunlight and can overcome many problems associated with sunlight exposure. A xenon lamp illuminates a wide range, while a laser beam illuminates a narrow area. Near-infrared (NIR) (700–810 nm), red (600–700 nm), yellow (550–600 nm), green (490–550 nm), blue (400–490 nm), and ultraviolet A (UVA) (330–400 nm) light have been applied for APDT [14]. A broad range of light has been used for APDT; however, longer wavelengths are preferred because of the deeper tissue penetration [15].

LEDs have become attractive light sources because they are easy to operate, safe, and inexpensive. Moreover, some innovative methods have been developed as alternatives to classic light sources. For example, wearable light-emitting fabrics facilitate the delivery of total light over a much longer time with a much lower power density, overcoming the problem of depletion of the available oxygen supply by decreasing the oxygen consumption rate [14]. The sufficient intensity of light for antibacterial photosensitization ranges from 5–1000 W/m^2^, as higher light intensities potentially result in thermal problems [16]. The exposure time varies from seconds to minutes, depending on the light intensity [16].

### 2.2. Oxygen

PSs and light irradiation are the main mechanisms of photodynamic therapy (PDT), and the ROS generated and the singlet oxygen (^1^O_2_) converted from molecular oxygen by PSs are responsible for bacterial damage [17]. Singlet oxygen damages organelles and causes programmed cell death in human cells [17].

Molecular oxygen (O_2_), a nonpolar small molecule that diffuses across biological membranes, is used by aerobic organisms for oxidation and respiration [18]. After O_2_ passes through the membranes, oxidative phosphorylation and adenosine triphosphate generation occur, and oxygen is reduced to produce energy [18]. Respiratory flavoenzymes are the main catalytic redox cofactors that participate directly in ROS formation by transferring ē to O_2_ to produce superoxide (O_2_^●−^) and H_2_O_2_ [18]. Afterwards, a Fenton reaction, in which H_2_O_2_ is oxidized with the available ferrous iron (Fe^2+^) to generate OH^●^, occurs [18]. Sodium azide (NaN_3_) and histidine are ^1^O_2_ quenchers, and thiourea and dimethyl sulfoxide are free radical scavengers that have been used in photodynamic inactivation therapies [19].

**Figure 1:**
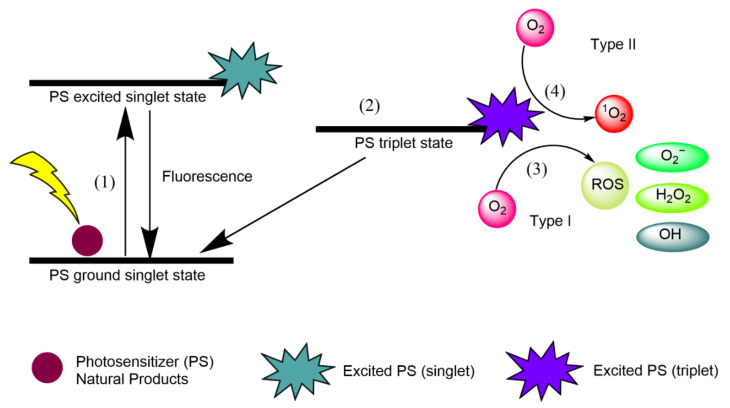
Type I and type II reactions in APDT. (1) Excitation from ground singlet state; (2) intersystem crossing into the triplet excited state; (3) Type I reaction; (4) Type II reaction.

Singlet oxygen (^1^O_2_) is an extremely short-lived and reactive form of oxygen that is involved in photochemical reduction processes. In type I reactions, electrons are stripped from biological macromolecules and ^•^OH is transformed into hydroxide ions [1]. Superoxide dismutase (SOD) converts O^•−^ into HO and O, and H_2_O_2_ participates in the Fenton reaction, resulting in the homolytic fission of the oxygen–oxygen bond in H_2_O_2_ to yield a hydroxide ion [1]. In type II reactions, ^1^O_2_ interacts with double bonds, sulfur moieties, and aromatic components of macromolecules in Diels–Alder cycloadditions [1]. Type I and type II reactions involving natural PSs are illustrated in Figure 1, and each step is explained below.

(1) Natural product PSs are converted from the ground singlet state into the excited singlet state when exposed to a specific wavelength of light.
(2) If the PS in the excited singlet state does not return to the initial ground state, it can be subjected to intersystem crossing into the triplet excited state.
(3) A type I reaction comprises transferring a hydrogen atom from PS to an organic molecule to form radicals, and the reduced PS interacts with oxygen through a redox reaction, forming ROS and a superoxide anion radical (O_2_^•−^) [20].
(4) The type II reaction comprises direct energy transfer from the activated PS to molecular oxygen to form singlet oxygen (^1^O_2_) and is simpler than the type I reaction.

### 2.3. Photosensitizers (PSs)

PSs play an important role in photodynamic reactions as absorbers of light energy [13]. PSs are divided into three subgroups, namely, first-, second-, and third-generation PSs. Water-soluble porphyrins called “hematoporphyrins” are characterized as first-generation PSs, and methylene blue, toluidine blue, photosense^®^, Foscan^®^, and 5′-aminolevulinic acid (ALA) are examples of second-generation PSs. They possess a higher singlet oxygen quantum yield, chemical purity, and selectivity than first-generation PSs [7]. Third-generation PSs have been investigated recently with the main aims of reducing damage to healthy cells and increasing bioavailability. These systems generally consist of drug delivery systems, gene engineering-based technologies, or monoclonal antibody receptor combinations.

An ideal PS should:

- Have a strong absorption peak in the red to near-infrared spectral region (between 650 and 800 nm) [15];
- Possess a substantial triplet quantum yield leading to good ROS production upon irradiation [21];
- Have high tissue selectivity [22];
- Exhibit no dark toxicity [23];
- Have ideal solubility to maintain lipophilic ability to cross the phospholipid membrane and prevent self-aggregation [24];
- Exhibit high stability under storage conditions [25];
- Kill microorganisms sufficiently without damaging eukaryotic host cells [26];
- Display optimal absorption, distribution, metabolism, and excretion (ADME) [24];
- Have a small size to enable microbial membrane permeation [2]; and
- Have low manufacturing costs [23].

## 3. APDT Targeting Diseases and Organisms

APDT targets many infectious diseases; for instance, *S. aureus* infections of the skin, soft tissue, and bloodstream, which are generally considered life-threatening, can be treated with APDT [27]. Many previous in vitro studies proved that APDT kills a broad spectrum of pathogenic microorganisms. The cellular structure and organization of microorganisms affect the efficiency of APDT. For instance, the different cellular structures of Gram-positive and Gram-negative bacteria influence the effects of APDT. The application of APDT to some important and well-known antibiotic-resistant microorganisms is also addressed in this section.

### 3.1. Target Components of Pathogenic Cells

Targeting vital components in microorganisms is a strategy for enhancing the efficiency of APDT. Target components of pathogenic cells related to compartments of microorganisms related to cell death have been identified. DNA damage in targeted microorganisms caused by PSs and light results in the breakage of the plasmid supercoiled fraction into single- or double-stranded DNA and is not the primary cause of cell death [28]. Membrane damage and the subsequent increased permeability, alteration of cytoplasmic membrane proteins, disturbed cell wall synthesis, and potassium ion loss are the other suggested causes of cell death [28]. In addition, APDT is also known to damage bacterial virulence factors, and Hamblin and Hasan [28] suggested naming APDT “antivirulence factor therapy”. DNA and RNA damage in target pathogens is also achieved by oxygen-independent antimicrobial photoinactivation using natural PSs. For instance, a natural compound, psoralen, generates interstrand DNA and RNA crosslinks and prevents replication and DNA synthesis [29].

### 3.2. Gram-Positive Bacteria

Gram-positive bacteria are the main targets of APDT and the subjects of many APDT studies. *S. aureus* shows resistance to widely used antibiotics [30]. Paramanantham et al. reported that APDT with the PS malachite green inhibits the growth of *S. aureus* biofilms by up to 80% [27]. *Streptococcus mutans*, which is responsible for forming dental biofilms, is also effectively suppressed by APDT [31]. *E. faecalis,* which causes endodontic infections, is another example of the use of APDT against Gram-positive bacteria [32]. Many isolates of *E. faecalis* are known to be resistant to ampicillin and result in a high incidence of vancomycin-resistant *Enterococcus faecium* infections, but APDT might be an effective treatment against such drug-resistant strains of *E. faecium* [32,33].

### 3.3. Gram-Negative Bacteria

APDT can also be applied effectively to kill Gram-negative bacteria. *Escherichia coli* is the most commonly studied Gram-negative bacterial target of APDT. As *Pseudomonas aeruginosa* is a biologically versatile organism that survives in both normoxic and hypoxic environments, it causes severe disease even in low-oxygen environments [34]. Abdulrahman et al. [35] reported that exposure to curcumin with light effectively treats *P. aeruginosa* by causing the downregulation of quorum sensing signaling. Alam et al. [36] reported that hypericin and ampicillin cotreatment with orange light effectively killed *P. aeruginosa.* As *Helicobacter pylori* directly affects the human gastric tract and causes associated disease symptoms such as diarrhea, nausea, and epigastric pain, the bacteria have been treated effectively by APDT [37], but as the stomach is an internal organ, some endoscopic techniques using laser probes are required [38]. Morici et al. [39] developed a novel LED device and tested its efficiency with a porphyrin PS to inhibit *H. pylori* by APDT. *Mycobacterium tuberculosis*, which is closer to Gram-negative bacteria, causes tuberculosis and shows high resistance to antituberculosis drugs and other injectable drugs due to its distinct and rigid cell envelope structure that forms an outer layer called the “capsule”, an outer membrane consisting of mycolic acid, several distinctive lipids, and an asymmetric cytoplasmic membrane; it is also considered a target of APDT [40]. Sung et al. [41] studied the effects of a chlorin derivative, PS, on *M. tuberculosis*.

### 3.4. Fungi and Oomycetes

APDT has also been used as an antifungal treatment. For instance, *Candida* sp. can occur in either a commensal or parasitic form, and the topical application of antifungals is insufficient for some cases, requiring complementary treatment. Many APDT studies related to fungal targets have been conducted. *Trichophyton mentagrophytes, Trichophyton tonsurans*, *Microsporum cookei*, *Microsporum gypseum*, *Microsporum canis*, *Epidermophyton floccosum*, *Nannizia cajetani*, *Metarhizium anisopliae*, *Aspergillus nidulans*, *Aspergillus fumigatus,* and *Fusarium* sp. have been treated using APDT with ALA, methylene blue, and many other PSs [1,42,43]. In addition, Zambounis et al. [42] investigated the biological effects of fagopytin and hypericin PSs on an oomycete named *Phytophthora citrophthora*. The pathogenic potential of *P. citrophthora* zoospores has been reported in many studies due to its destructive damage to crops worldwide [42]. When exposed to light and these PSs, the zoospores failed to cause necrotic lesions and penetration events, implying decreased virulence [42].

### 3.5. Viruses

Viral infections are treated with antiviral drugs such as acyclovir. However, after a certain time, drug resistance might be observed and cause the treatment to become inefficient [43]. Many studies are investigating the possible application of APDT for viruses to overcome this issue. Monjo et al. [44] indicated that the use of LEDs with orthoquin compounds derived from *Polygonum cuspidatum* is significantly effective against herpes simplex virus. Additionally, some recent studies have investigated the use of APDT against Coronavirus disease 2019 (COVID-19) with methylene blue and porphyrin PSs [45]. Moreover, many studies have shown that porphyrins and ALA PSs induce the highly efficient inactivation of T4-like bacteriophages [9].

### 3.6. Mosquitoes

Photodynamic processes combined with singlet oxygen and ROS are the main chemical weapons used against insects and photodynamic processes have been applied to pesticide-resistant mosquitoes [46]. The accumulated PS in the pest body causes lethal photochemical reactions when the pest is exposed to visible light [46]. Rose bengal-induced phototoxicity was 100 times more effective than chlorpyrifos, a commercially available insecticide, against *Culex pipiens* larvae [46].

### 3.7. Plants

Siewert and Stuppner [47] hypothesized that many isolated bioactive phytochemicals have hidden photoreactive potential. As the chromophore part of photosynthesis molecules is responsible for light absorption at certain wavelengths, natural products exhibit high photoreactive antimicrobial activity [47]. PSs are produced by plants as a defense mechanism, and this defense mechanism was reported to combat the decline of many plant species, such as banana. Banana with red dye in its root easily combats banana plant diseases, fending off the so-called banana-geddon [47]. Moreover, the introduction of an exogenous PS to plant cells may cause significant damage to plant tissues. Therefore, unwanted plants can be controlled by exogenous PSs. Compared to conventional herbicides, PSs are safer for birds and other wild animals. As ALA is a precursor of tetrapyrrole compounds, it has been used as a photodynamic herbicide to kill plants [48].

### 3.8. Parasitic Protozoa

The parasitic euglenoid *Trypanosoma cruzi* causes Chagas disease [49]. This disease can be transferred to humans by triatomine bugs and sometimes orally. Pheophorbide was used as a PS to kill the euglenoid through APDT and treat this disease [49]. Anthraquinones and violet-blue LEDs were applied to *Leishmania amazonensis* to treat vector-borne disease cutaneous leishmaniasis [50].

## 4. Preclinical and Clinical APDT Studies

In addition to in vitro screening studies, preclinical and clinical APDT trials have been performed to evaluate the efficacy and side effects of APDT methods. For preclinical studies, animal models are preferred; for clinical studies, the effect of APDT is directly measured in humans.

### 4.1. Preclinical Animal APDT Studies

Generally, before the application of APDT in human clinical trials, many in vivo studies using mammals (e.g., rats and mice) are performed to broaden the range of diseases for treatment, such as *H. pylori*, leishmaniasis, tuberculosis, osteomyelitis, nasal infections, oral infections, wounds, and burns [14]. After assessing the in vitro antimicrobial activity of APDT in cultured microorganisms without nontargeted eukaryotic animal cells, the selectivity of APDT is tested in a coculture of pathogenic microorganisms and host cells. Cell culture assays are easily performed techniques with no ethical concerns regarding the use of animals. Therefore, many studies use cell culture systems for the initial screening of APDT efficiency [51]. Rupel et al. [52] investigated the APDT potential of a curcumin-based PS against *P. aeruginosa* in human keratinocyte cell lines.

In addition to cell culture, in vivo animal models can be used to evaluate the therapeutic effects and side effects of APDT. Many preclinical APDT studies are being performed to determine the most suitable PS, concentration, and exposure time to treat various diseases. Many studies in rats and mice have confirmed the efficiency of APDT. For instance, the work of Sahu et al. [53] indicated that diabetic wounds in mice are healed by APDT with a topical application of PS. Skin abrasions, burn infections, and excisional wounds are other common applications of mouse models [54]. Mouse models have been used to evaluate the tissue specificity of PS application and its ADPT effect. The antifungal activity of curcumin was investigated in a murine model by evaluating histology, immunohistochemical staining, and DNA damage [55]. In addition to mouse models, many other organisms have been used for in vivo studies, such as the nematode *Caenorhabditis elegans* and the wax moth *Galleria mellonella*.

*C. elegans,* a self-reproducing nematode with a short life span and a transparent body, is a preferred animal model in many APDT studies because of its easy visualization under a microscope and lack of ethical concerns [36,56,57,58,59,60]. Our group was the first to document the APDT effects of natural PSs, hypericin, and plant extracts on *C. elegans* infected with various pathogenic bacteria [36,56]. After APDT, *C. elegans* survived without any significant side effects and the growth delays induced by pathogen infections were reversed [36,56].

**Figure 2:**
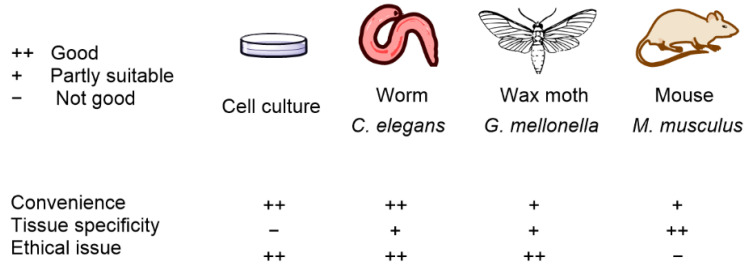
Model organisms and their main properties in APDT testing.

The wax moth *G. mellonella* is another model organism used to identify the efficiency and toxicity of antimicrobial agents and is likewise a preferred model organism for avoiding ethical concerns related to the use of mammals. Huang et al. [61] reported that *G. mellonella* is a leading infection model organism, especially in the field of PDT against *Candida albicans* and *S. aureus. G. mellonella* studies investigate light penetration, and Figueiredo-Godoi et al. [62] found that light is distributed 0.27–2.45 mm below cuticle *G. mellonella* larvae. Model organisms and their main properties are summarized in Figure 2.

### 4.2. Clinical APDT Trials

Many clinical studies on the treatment of nonhealing ulcers, dental infections, acne, gastric infections, and many other diseases using APDT have been performed [63]. Oral decontamination of orthodontic patients was achieved by APDT using blue light and curcumin and promoted with sodium dodecyl sulfate surfactant [64]. In addition, Ivanaga et al. [65] performed curcumin-based APDT as an adjunct therapy in combination with scaling and root planing to treat residual pockets in patients with diabetes. Additionally, *Staphylococcus* spp. and Enterobacteria were eliminated from the mouths of patients with AIDS using curcumin and blue light [66]. In addition to those applications, Song et al. [67] used chlorophyll a and Nicklas et al. [68] used ALA to treat patients with acne vulgaris. Chlorophyll a was applied with light at intensities of 6000 and 6500 W/m^2^ for 30 min, and ALA was applied with a light intensity of 685 W/m^2^. Both APDTs induced a substantial reduction in acne inflammation lesions in the patients [67,68]. Zangirolami et al. [69] studied the photoactivation of curcumin-functionalized endotracheal tubes using an optical fiber. ALA PDT was also used to inactivate human papillomaviruses and treat condyloma acuminatum, a disruption of the epidermis with hyperkeratosis [70].

Some commercial PS drugs are derivatives of natural PS and are also undergoing clinical trials for use in APDT. Photodithazine^®^, a chlorin-derived PS, was used in denture stomatitis [71] and against *C. albicans* [72] in clinical studies. Wiegell et al. [73] evaluated the potential application of the methyl aminolevulinate-based drug Metvix^®^ for acne treatment and found similar effects to those of ALA. Another ALA-based drug named Levulan^®^ was clinically tested against acne and shown to have higher efficiency than commercial acne topical cream [68].

## 5. Natural PSs in APDT

**Figure 3:**
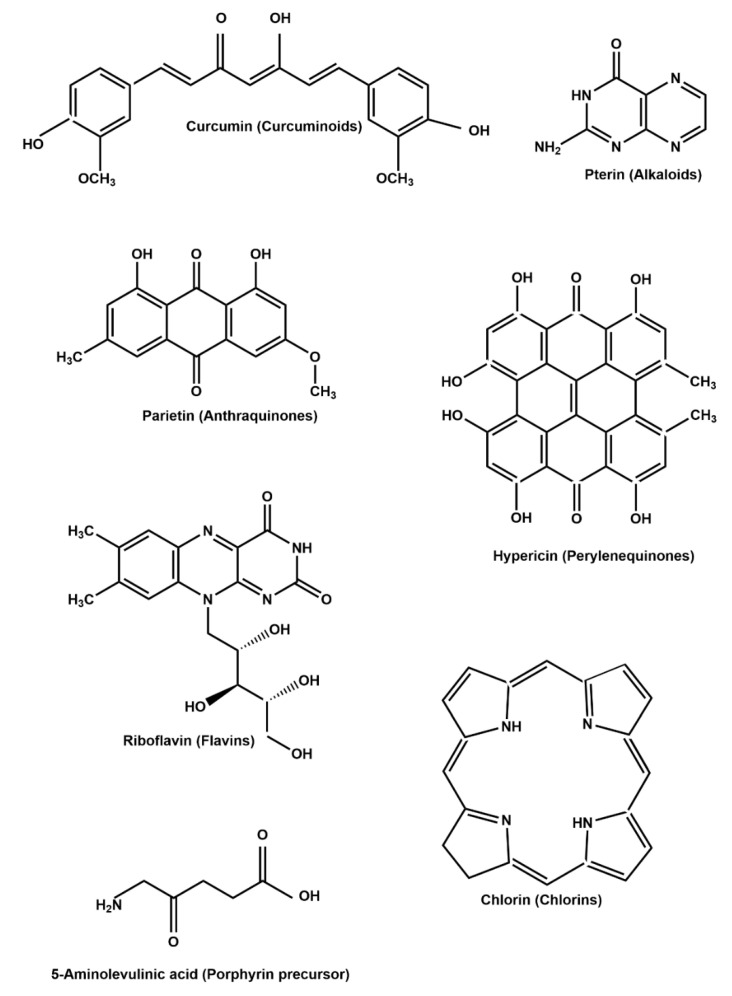
Common natural PSs used in APDT.

Plant extracts enriched with chlorophyll exert encouraging photodynamic effects due to the high quantum yield of singlet oxygen (^1^O_2_) since they show high absorption under visible light [74]. Natural PSs are extracted from plants, fungi, and bacteria. Both natural compounds and extracts have been used as PSs for APDT. For instance, Bonifácio et al. [75] examined *Curcuma longa* extract and found that APDT using *C. longa* is effective against *Listeria innocua* biofilms. The main chemical structures of common PSs are shown in Figure 3. Representative natural PSs composed of compounds and extracts and their application in APDT are summarized in Table 1 and Table 2.

### 5.1. Curcuminoids

Curcumin is mainly isolated from the rhizome of turmeric, *C. longa,* and has a wide range of biological activities, such as antiviral, anti-inflammatory, antitumor, and antibacterial properties [24]. Turmeric roots have been commonly used for food and therapeutic purposes in Asian countries for centuries and have broad applications in APDT studies [76]. Curcumin is activated by blue light, as its absorption wavelength ranges from 405 to 435 nm [23]. As curcumin has a hydrophobic structure, some modifications are required for its use as a PS in PDT applications [23]. Representative examples of APDT with curcumin are summarized in Table 1. Curcumin exerted an inhibitory effect on the growth of Gram-negative, Gram-positive bacteria, and *Candida* sp. in food-related diseases, periodontitis, wound healing, and multidrug resistance. Curcumin concentrations ranging from 0.5 µM to 6.1 mM have been effectively used in APDT studies [35,77,78,81,82,83,84,86,87,88,89,90]. In addition, mosquito larvae are controlled by curcumin exposure for 120 min at a light intensity of 220 W/m^2^ [91].

### 5.2. Alkaloids

Light-dependent changes in alkaloids, the second largest group of natural products, were first suggested in 1888 [47]. Natural photoactive alkaloids are classified into five categories: quinoline-based alkaloids, pterins, benzylisoquinolines, beta-carbolines, and indigo alkaloids. Quinoline-based alkaloids (palmatine hydrochloride and berberine) are known to possess photocytotoxic activity, and pterins, such as 6-hydroxypterin in butterflies, are also known for their PS properties [47]. The indigo PS from the plant *Indigofera tinctoria* shows photoreactivity against both Gram-positive and Gram-negative bacteria [47]. Treatment with pter-in-6-carboxylic acid alkaloids with a 31 W/m^2^ light intensity was effective against *Klebsiella pneumoniae* [85].

### 5.3. Anthraquinones

Anthraquinones, which are commonly classified into monomeric and dimeric anthraquinones, are produced by the acetate/malonate pathway and the shikimic acid/mevalonate pathways [47]. Emodin (excitation wavelength; 434 nm), rhein (437 nm), rubiadin (410 nm), physcion (438 nm), carminic acid (494 nm), and pupurin (515 nm) are representative anthraquinones that are mainly isolated from plants [47]. The root of the plant *Polygonum cuspidatum* used in traditional Chinese medicine contains rhein, emodin, and physcion anthraquinone [47]. *Heterophyllaea pustulata* Hook f. (Rubiaceae) contains ten different types of anthraquinones, namely, soranjidiol, soranjidiol 1-methyl ether, rubiadin, rubiadin 1-methyl ether, damnacanthal, damnacanthol, heterophylline, pustuline, 2-hydroxy-3-methyl anthraquinone, and (S)-5,5′-bisoranjidiol, which have the potential to be used as PSs [103]. Aloe emodin is a common anthraquinone that is used for APDT. Aloe emodin has been reported to be effective against *S. aureus*, *E. coli*, *A. baumannii*, and *C. albicans*. APDT with aloe emodin resulted in a considerable reduction in the number of bacteria and fungi [97,98,99]. Moreover, Comini et al. [96] investigated the APDT potential of parietin, an anthraquinone PS, with a 1000 W/m^2^ light intensity.

### 5.4. Perylenequinones

Herb buckwheat (*Fagopyrum esculentum*), aphids such as *Hormaphis* sp., and the protozoan *Blepharisma* sp. are the main sources of perylenequinones [47]. Hypocrellin A and hypocrellin B are perylenequionone compounds isolated from *Hypocrella bambusae* [24]. Under acidic conditions, hypocrellin A is converted into hypocrellin B, and both compounds have photodynamic activity, have low dark toxicity, and generate large amounts of singlet oxygen [24]. Hypocrellins have photodynamic antifungal activity against *C. albicans* after 30 min of exposure to light (400–780 nm) [95]. Hypericin isolated from *Hypericum perforatum* (St. John’s wort) has been used in folk medicine due to its antiviral, antitumor, antidepressant, and antibiotic properties [24]. Hypericin localizes in cytosolic organelles of *Candida* sp. and is effectively used for fungal photoinactivation. Hypericin has a high quantum yield capacity with slow photobleaching [104]. Kashef et al. [104] evaluated hypericin with the antibiofilm agent acetylcysteine. Treatment with both hypericin and acetylcysteine exerted a synergistic effect on *S. aureus* biofilms, as acetylcysteine made the biofilms more susceptible to phototoxicity [104]. In addition, the photoactivation of hypericin to combat food-borne pathogens and acne was investigated, and hypericin had a substantial ability to inactivate microbial pathogens. However, hypericin exhibits poor water solubility and absorbs light at 590–600 nm [24]. Because of its hydrophobic nature, liposomes, micelles, and nanoparticles may be applied for hypericin delivery. Hypericin (10 µM) with 1 h of exposure to orange light (590 nm, 150 W/m^2^) showed potent antimicrobial activities against the Gram-positive bacteria *S. aureus* and MRSA, and the fungus *C. albicans*. However, even after 3 h of exposure to orange light (150 W/m^2^), hypericin did not inhibit the growth of Gram-negative bacteria *P. aeruginosa* because of the poor cellular uptake of hypericin by Gram-negative bacteria [36]. Therefore, additional technology is required to overcome the limitation of APDT with a natural PS.

### 5.5. Flavins

Riboflavin, vitamin B2, is a well-known flavin that is present in a broad range of organisms, such as human tissues, plant leaves, mushrooms, and eggs [24]. Riboflavin, which has two absorption peaks in the UVA (360 nm) and blue (visible, 440 nm) regions, has been used in APDT [23,105]. It also has a high quantum yield and substantially inhibits the growth of antibiotic-resistant bacteria such as enterohemorrhagic *E. coli* and MRSA [24]. Riboflavin (100 µM) with 90 min of exposure to light (460 nm, 150–580 W/m^2^) successfully inactivates *Listeria monocytogenes* on smoked salmon fillets [100].

### 5.6. Porphyrins, Chlorins, and Bacteriochlorins

Natural molecules such as chlorophyll, heme, and cobalamin are known groups of tetrapyrrolic macrocycles and are considered “pigments of life” [106]. Porphyrin and chlorins are the most commonly used PSs for PDT studies. Porphyrin has an absorption band in the region of 400 nm (Soret band) and other small bands in the region of 630 nm. Chlorin-type PSs have a strong absorption band in the violet–blue region (380–450 nm, B or Soret band) and a moderate band in the red region (600–700 nm, Q band) [106]. Most bacteriochlorins show a strong absorption in the NIR region but exhibit low stability and conversion to chlorin and/or porphyrin precursors [106,107].

According to some studies, cationic porphyrins are particularly effective at photoinactivating bacterial species on tissues and surfaces, and thus porphyrin-based APDT provides efficient bacterial removal without inducing antimicrobial resistance and without affecting the microflora of the body [108]. Porphyrins are preferred organic PSs due their strong ^1^O_2_ generation efficiency and excellent fluorescence properties [109]. However, their poor water solubility and low extinction coefficient in the NIR region limit their usage in PDT studies. ALA is a porphyrin precursor with good hydrophilicity, allowing it to accumulate in tissues with high efficiency [24]. ALA (50–300 mM) was used effectively against trichosporonosis with 10–30 min of exposure to light (600 W/m^2^) [92].

Chlorins are derived from chlorophyll a and show maximum absorption between 650 nm and 700 nm [24]. This region allows them to penetrate deeper tissues. Chlorins are also porphyrin derivatives with extra hydrogen atoms in the pyrrole ring [24]. Chlorins generate a large amount of singlet oxygen, but their poor solubility and instability in the presence of light, acids, and bases limit their application [24]. However, many chlorin PS applications in the microbial control of seeds, plant disease, and multidrug resistance have been reported [97,106,107]. Chlorophyllin sodium salt at low (1 µM) or high (15 mM) concentrations showed effective antimicrobial properties upon exposure to light intensities of 96 and 148 W/m^2^ [101,102].

Bacteriochlorophylls are tetrahydroporphyrins with two reduced pyrroles positioned on opposite sides of the macrocycle that are present in some photosynthetic bacteria [107]. Chlorophyll a absorbs at 662 nm, chlorophyll b absorbs at 644 nm, and bacteriochlorophylls a, b, and g are characterized by strong absorption at 772 nm, 794 nm, and 762 nm, respectively [107]. Bacteriochlorophylls have also been applied for the photodynamic inactivation of microorganisms [107].

### 5.7. Natural Extracts

In addition to single compounds from natural sources, natural extracts themselves have also been used as PSs in many studies. For example, biologically active extracts possess high antifungal properties and retard the reproduction and growth of plant pathogenic fungi and their oomycetes through light-dependent excitation [110]. Representative examples of natural extracts used in APDT are shown in Table 2.

Gonçalves et al. [111] studied the effects of a *Bixa orellana* extract on Gram-negative biofilms, and performed clinical studies and reported that 20 s of exposure to 1540 W/m^2^ light with the extract reduced halitosis in a clinical trial. Andreazza et al. [110] investigated APDT using a *Guatteria blepharophylla* extract to control a broad range of microorganisms. The authors reported that 5 min of exposure to 900 W/m^2^ light (660 nm) with the extract showed an APDT effect. Dascalu et al. [112] studied frankincense essential oil and mixed essential oils to control microorganisms in oral cavities via APDT. Saitawee et al. [113], Hormdee et al. [114], and Lee et al. [115] investigated APDT with *Curcuma longa* or *Curcuma xanthorrhiza* extracts against periodontitis, adjunctive treatment, and planktonic biofilms upon exposure to 840 to 12,000 W/m^2^ light intensities. Moreover, Saint John’s wort extract-mediated ADPT inhibited oral biofilms [116]. Saint John’s wort extract was also studied against human enteric virus under solar irradiation [117]. Giacone et al. [118] studied the photodynamic effects of *Tagetes minuta* extracts on *Candida* species. In addition, APDT approaches using *Hibiscus sabdariffa* extract in the sanitation of food [119], *Indigofera truxillensis* extract against multidrug-resistant microorganisms [110], *Porophyllum obscurum* (Spreng.) DC. extract against oropharyngeal candidiasis [120], and *Eucalyptus microcorys* leaf extract against *E. coli* were investigated [121]. Our group also reported the APDT effects of a *Tripterygium wilfordii* extract and its PS-enriched fraction on various bacteria and fungi, especially skin pathogens [56]. In that study, the APDT effects were also evaluated on the model nematode *C. elegans* treated with pathogenic bacteria, and APDT apparently ameliorates symptoms in *C. elegans* infected with various pathogenic bacteria [56]. The *T. wilfordii* extract and its PS-enriched fraction (20 µg/mL) with a 10 min exposure to red light (660 nm, 120 W/m^2^) potently inhibited the growth of various Gram-positive bacteria, such as *S. aureus*, MRSA, *S. epidermidis*, and *Streptococcus pyogenes*. In contrast, PS with a 30 min exposure to red light (660 nm, 120 W/m^2^) did not inhibit the growth of the Gram-negative bacterium *Aeromonas hydrophila*. The different APDT effects on Gram-positive and Gram-negative bacteria are probably due to the differences in the uptake of PSs by bacterial cells. The studies imply that some additional technology is required to overcome the limitation of APDT with natural PSs, including a low antimicrobial effect on Gram-negative bacteria, which is described specifically in the next sections.

## 6. Synthetic Derivatives of Natural PSs

Different parameters related to synthetic derivatives of natural PSs are described in this section. PSs are obtained by natural extraction or by semisynthetic or synthetic methods. For instance, chlorin-based PSs have been produced from chlorophyll using several methods:

- Direct isolation of natural chlorophylls from plants [124,125];
- Synthesis of chlorin-based PSs by hydrogenation, annulation, cycloaddition, breaking, and mending of porphyrin precursor [126,127,128,129];
- Semisynthesis of chlorin-based PSs from natural chlorophylls [130]; and
- De novo synthesis of gem-dialkylchlorins using a reduced ring of the acyclic precursors of chlorin [106,131].

Both natural and synthetic PSs can be used for APDT. Natural PSs themselves have been used directly in APDT without the additional development of chemical synthesis processes to produce synthetic PSs, which enables the development of new APDT methods with a lower cost. Many natural PSs originate from edible plants and do not require harmful organic chemicals for manufacturing synthetic PSs; therefore, natural PSs are consumer-intimate and environmentally friendly. However, natural PSs sometimes possess a low triplet quantum yield upon irradiation, low solubility in aqueous solution, and poor ADME properties. Therefore, synthetic derivatization of natural PSs with better APDT properties is important. However, the discovery of new natural PSs is of course essential for the development of new synthetic PSs.

Synthetic derivatives of natural PSs are important mimics of bioactive compounds with enhanced properties. Many studies have investigated synthetic derivatives of natural PSs, and their application parameters are summarized in Table 3. For instance, phthalocyanine derivatives in the presence of metal atoms, such as Zn, Al, and Si, yield a long T1 lifetime and a high ^1^O_2_ generation quantum yield, and these PSs have sufficient photophysical and photochemical properties [109]. In addition, Seeger et al. [108] evaluated the APDT activities of two different tetracationic porphyrins (H_2_TMeP (free-base porphyrin) and ZnTMeP (zinc(II) derivative porphyrin)) against Gram-positive and Gram-negative bacteria that are commonly observed in canine otitis. Photoinactivation of both Gram-positive and Gram-negative bacteria was achieved. The best results were obtained against *P. aeruginosa* and *Proteus mirabilis* using H_2_TMeP, which achieved complete bacterial inactivation after 60 min of exposure, while ZnTMeP reached the maximum bacterial inactivation at 90 min [108]. Semisynthetic bacteriochlorins are produced by changing the centrally coordinated metal ion in the bacteriochlorophyll macrocycle: Mg^2+^ is replaced with Zn^2+^, Ni^2+^, Cu^2+^, Pt^2+^, and Pd^2+^ [107]. Synthetic derivatives of bacteriochlorins maintain stability with hydrophilic or amphiphilic properties.

The perylenequinone derivative hypericin-glucamine is another example of a synthetic PS derivative that promotes periodontal repair [132]. Bresolí-Obach et al. [133] reported that a phenalenone PS showed significant photostability and phototoxicity against Gram-positive bacteria. Additionally, in that study, a triphenylphosphonium derivative PS selectively killed Gram-positive bacteria. In another study, antiviral photodynamic activity was observed for porphyrin-derivative PSs [134]. Guterres et al. [135] reported that cationic porphyrin derivatives showed higher singlet oxygen production with higher photostability than anionic porphyrin derivatives. Moreover, low concentrations of cationic porphyrin derivatives showed very strong antimicrobial photodynamic activity against MRSA. Light intensities ranging from 250 to 1667 W/m^2^ were effective against *Mycobacterium* species and MRSA [136]. Biyiklioglu et al. [137] showed that phthalocyanine derivatives exerted a favorable antibacterial effect on both Gram-positive and Gram-negative bacteria. Phthalocyanine derivatives exhibit a photodynamic fungicidal effect [138] and a strong APDT effect against Gram-negative bacterial biofilms [139]. Phthalocyanine derivatives have also shown strong photodynamic antibacterial activity against *S. typhimurium* [140]. Moreover, chlorin derivatives showed high efficiency against a broad range of microorganisms under blue light (56 W/m^2^) [141], and bacteriochlorin derivatives with red light (278 W/m^2^) were highly effective against Gram-negative bacterial biofilms [139].

## 7. Current Limitations of APDT

APDT can inactivate a broad spectrum of bacteria. However, the properties of PSs and light sources affect the efficiency and side effects of APDT. Poor water solubility and aggregation are the main problems associated with many traditional PSs [17]. Moreover, ultraviolet light has poor penetration and high cytotoxicity [17]. Neutral or anionic PS molecules efficiently bind to Gram-positive bacteria and photodynamically inactivate them, but they are often inactive against other microorganisms, including Gram-negative bacteria [143].

### 7.1. Limitation of APDT against Gram-Negative Bacteria

The localization of PSs to target microorganisms depends on many factors, such as the molecular size, charge, lipophilicity, concentration of PSs, and cell wall structure of the target microorganisms [26]. Weak interactions between PSs and Gram-negative bacteria limit the application of APDT [144]. The outer membrane of Gram-negative bacteria comprises an asymmetric bilayer consisting of phospholipids and lipopolysaccharides. The phospholipid structure of Gram-negative bacteria is composed of approximately 15% phosphatidylglycerol, 80% phosphatidylethanolamine, and 5% cardiolipin [145]. Small hydrophilic drugs, such as β-lactams, use pore-forming porins to obtain access to the cell interior, whereas macrolides and other hydrophobic drugs diffuse across this lipid bilayer [145].

The Gram-negative bacterial outer membrane consists of a phospholipid bilayer with hydrophilic surfaces and a lipophilic core [10]. Hydrophilic molecules pass through the phospholipid bilayer, while lipophilic molecules are retained in the bilayer. Some molecules with both lipophilic backbones and polar/charged flanks pass through the lipid bilayer due to their amphipathic nature [10]. For instance, curcumin and hypericin are the two main PSs known for their amphipathic nature. Many studies have shown the inefficiency of APDT against Gram-negative bacteria. For instance, Alam et al. [36] mentioned that the membrane structure of *P. aeruginosa* resulted in a low efficiency of APDT, and they suggested the application of membrane-damaging antibiotics together with PS to facilitate the uptake of the PS hypericin.

### 7.2. Limitation of Selectivity (Human Cells and Good Bacteria)

Targeted PDT is an important issue because PDT also damages unintended targets, such as human tissue and beneficial microorganisms, resulting in side effects [11]. APDT targets a broad spectrum of pathogens; however, it is nonselective regarding multiple molecular targets, such as proteins, lipids, and nucleic acids. In particular for cationic PSs, electrostatic interactions between mammalian cells and PSs have resulted in poor target selectivity [26,146]. The main goal of APDT is to kill pathogens without damaging human cells.

Chlorophyll derivatives are preferred to control pests and disease vectors because they are cost effective and environmentally friendly [147]. However, PSs introduced to the environment kill nontargeted larvae and eggs, in addition to other nontarget organisms. They pose potential risks to the whole environment if introduced to ecosystems. The application of PSs to humans can result in side effects such as photosensitivity. Human skin has some problems associated with PS sensitivity, and redness, swelling, and rare allergic reactions have been observed [63].

### 7.3. Limitations of Solubility and Light Penetration

The poor solubility of PSs is a major problem limiting the widespread application of APDT. This limitation potentially causes bioavailability problems and blocks the cellular uptake of PSs. Additionally, limited solubility might result in the susceptibility of PSs to hydrolytic degradation and even aggregation before interaction with the target cell. This aggregation causes aggregation-induced fluorescence quenching and low generation of ROS, resulting in a low APDT potency. Although light penetration is not a common problem in APDT, APDT is not effective against some deep lesions. For instance, *P. aeruginosa* lesions can reach up to 15 mm [139], and longer wavelengths (720–850 nm) should be considered for deeper penetration.

### 7.4. Limitation of Economic Efficiency and Quality Control

The purification of PSs from natural resources is time consuming and sometimes generates toxic waste [74]. Low-cost extracts may provide good APDT performance, while single-compound PSs are generally considered not cost effective. Single-compound isolation is a time-consuming process and requires special equipment. Economically efficient scaled up technology is required to produce single-compound PSs. Natural extracts with nonenvironmentally friendly extraction processes are expensive. Major active PS compounds present in natural extracts depend on many environmental factors, such as growth temperature, seasons, daily illumination, and many other exogenous factors, and the contents of active compounds significantly influence the effectiveness of ADPT. Therefore, chemical profiling analyses and the standardization of natural extracts are essential for the quality control of new APDT methods with natural extract PSs, which also increases the research and development costs for new ADPT methods.

Low-cost, high-performance APDT is a popular research topic, and many practical studies are required. Synthetic strategies may provide cheaper PSs with high efficiency compared to the extraction and purification of natural products. Many processes are being investigated in pursuit of lower prices. As maceration, sonication, and Soxhlet are well-known processes to extract natural sources, new technologies such as pressurized liquid extraction may overcome many disadvantages of other extraction processes [74].

## 8. Emerging Technologies and Solutions to Current APDT Limitations

The current limitations of APDT are altered by introducing new techniques to improve PS properties or by introducing new compounds. For clinical applications, the costs of new and conventional antimicrobial therapies should be considered, and emerging alternative methods are becoming increasingly important [26]. In addition, the development of new antibiotics is time consuming and is not efficient in many cases [5]. Therefore, some novel strategies have been developed to solve the current limitations of APDT.

### 8.1. Cell Surface Engineering for Enhanced Delivery and Solubility of PSs

Cell surface engineering is an increasing trend in the area of cell surface interactions with chemicals. Cell surface engineering potentially results in modified cell membranes with new functions related to hydrophobic interactions, electrostatic interactions, and covalent conjugations [148]. Jia et al. [148] facilitated hydrophobic interactions using cholesterol-assisted bacterial cell surface engineering, resulting in the high bacterial inactivation efficiency of APDT. The lipophilic nature of chlorophyll a and b and their photoactive degradation products, which promote self-aggregation and minimize APDT activity, are overcome by solubilization using triblock amphiphilic copolymers [74].

Polymer-based conjugates derived from cyclodextrins are well-known compounds that have been investigated to enhance APDT efficiency. These cyclodextrins have been used to encapsulate PSs and improve their physiological properties. In addition, the controlled ROS activity of PSs, such as increasing, decreasing, or switchable trends, and the response of PSs to environmental stimuli are controlled by cyclodextrins [149]. For instance, Ferro et al. [150] studied cyclodextrin and porphyrin PSs to strengthen their antimicrobial photosensitizing properties against MRSA. Castriciano et al. [151] investigated the combination of PSs and cyclodextrin for controlled delivery to *S. aureus* and *P. aeruginosa*. In addition to cyclodextrin, chitosan materials have also been applied to the fabrication of antimicrobial biomaterials [2]. Sharma et al. [152] studied PS-embedded cellulose films and found that composite polymer films facilitate the bacterial uptake of PS. Additionally, Contreras et al. [153] reported that PS-loaded fibers are photodynamically active conjugates. Chandna et al. [154] developed lignin hydrogels against *Candida* sp. to investigate the APDT potential and found that these hydrogels have potential for use in stimuli-responsive APDT. Moreover, Liu et al. [155] used a tannic acid coating to modify water-soluble chlorin-derivative PSs.

### 8.2. Protein Engineering for Increased Specificity

Antimicrobial peptides are another set of molecules that have been used in APDT. Antimicrobial peptides are natural peptides of 12–50 amino acids in length that rapidly kill various bacterial cells and selectively kill prokaryotes [156]. This selective killing is related to their positive net charge, and Freitas et al. [156] proved that an antimicrobial peptide named aurein 1.2 is feasible for use in APDT against a broad range of pathogens, such as *E. faecalis*, *E. faecium*, *S. aureus*, *A. baumannii*, and *E. coli*. Antimicrobial peptides induce rapid bacterial killing by enhancing membrane instabilities caused by the accumulation of PSs and antimicrobial peptide complexes and thereby rendering membranes vulnerable to light exposure [156].

Genetically engineered antimicrobial PSs maintain a better environment for the fusion of PS proteins with target peptides or antibodies and might promote the accumulation of PSs in cellular compartments [157]. Genetically encoded proteins provide improved photosensitization with selective killing and no dark toxicity. These genetically engineered proteins might release a sufficient amount of singlet oxygen to kill bacteria and enable sufficient cytoplasmic localization of PSs [158]. Flavin-binding proteins and green fluorescent proteins are two known examples of genetically encoded proteins. Hally et al. [159] described genetically encoded proteins, including fluorescent proteins and flavin-binding proteins, and emphasized that flavin-binding proteins can exhibit good photosensitizing properties for antibacterial photodynamic inactivation. In addition, Torra et al. [160] investigated the photosensitizing potential of a genetically encoded protein named Mini Singlet Oxygen Generator (MiniSOG), a flavoprotein that binds to flavin.

### 8.3. Enhanced PS Uptake Strategies via Electroporation and Chemicals

Reversible or irreversible cell membrane unsealing have been induced by electrical pulses, a process called electroporation [22]. This procedure has been used for the genetic transformation and transport of drugs through the membrane. De Melo et al. [161] indicated that electroporation increased the cellular uptake of the PS hypericin by both Gram-negative and Gram-positive bacteria. Electroporation solves the limited uptake of many PSs, such as hypericin, which shows limited water solubility. In addition to electroporation, calcium chloride and ethylenediaminetetraacetic acid (EDTA) treatments increase the cellular uptake of PSs by the target bacteria. Winter et al. [162] indicated that calcium chloride, a permeation inducer, enhanced Gram-negative bacterial inhibition. In addition, Tennert et al. [163] reported that EDTA, a chelating agent, inhibited biofilm formation by increasing the ability of PSs to penetrate and disintegrate bacterial biofilms. The combination of PSs with antibiotics also increases the efficiency of APDT. For instance, Pérez-Laguna et al. [164] investigated the efficiency of APDT when PSs were combined with the antibiotics mupirocin and linezolid. In addition, Alam et al. [36] indicated that cotreatment with the membrane-damaging antibiotic ampicillin resulted in increased membrane permeability to the PS hypericin and improved the effectiveness of APDT against Gram-negative bacteria.

### 8.4. Nanotechnology for Increased Solubility, Specificity, and Cost

Nanotechnology and natural PS combinations enhance the current efficacy of APDT and decrease side effects [12]. Nanoscale drug delivery is important due to its ability to transport hydrophobic products into the bloodstream. Moreover, functional group modifications improve the biochemical properties of nanoparticles, and nanosystems enable the controlled release of the delivered drug [165]. Nanoscale PS delivery systems increase treatment efficiency by minimizing the side effects of conventional APDT [22,165]. These nanoscale PS delivery systems might diminish phototoxicity, increase cellular uptake and provide biostability to PS compounds. Synthetic strategies can easily be applied to nanoscale molecules to improve their main properties.

**Figure 4:**
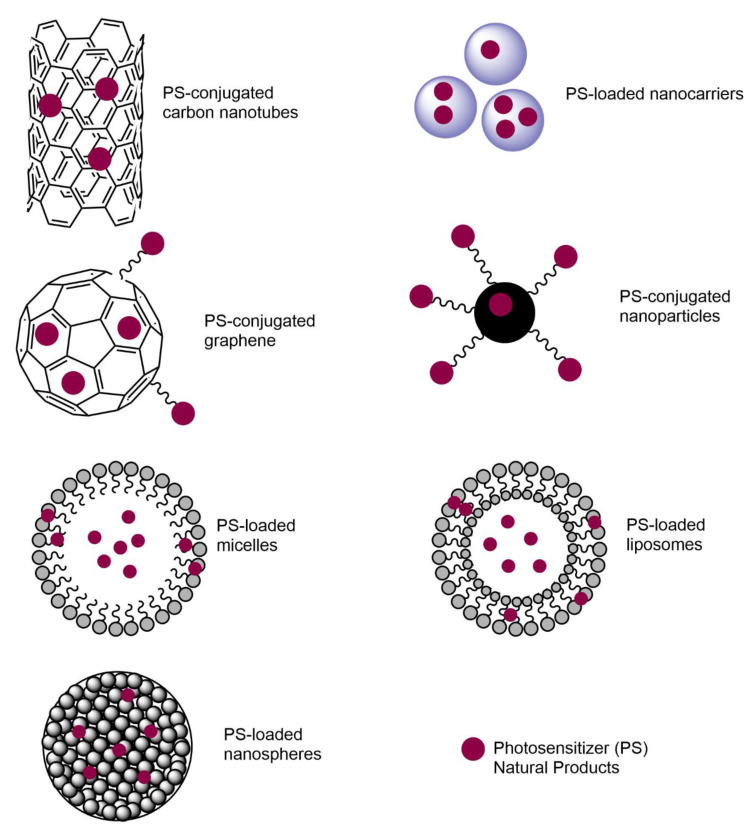
Nanotechnology-based PS structures that are used in APDT studies.

Nanoparticles smaller than 100 nm with an improved surface-to-mass ratio are important for many biomedical applications. When nanoparticles are used for antimicrobial purposes, high penetration into the bacterial membrane and high disruption of biofilm formation are observed. In addition, nanoparticles possess multiple antimicrobial mechanisms and carry antibiotics efficiently [5]. However, they have potential toxicity, as they show high reactivity due to the high surface area-to-mass ratio. Specific PSs have been designed for increased efficiency and to overcome the side effects of PDT on human cells. For instance, Zhuang et al. [17] designed and synthesized lysosome-targeting PSs that were applied in smaller doses to eliminate the toxicity with increased specificity toward Gram-positive bacteria. As lysosomes are known to be the primary degradative organelle, degrading macromolecules and organelles into amino acids, monosaccharides, and fatty acids, lysozyme-targeting PSs can be effectively used in clinical applications [17]. Hydrophobic PSs can be modified to a more water-soluble form by introducing HSO_3_^−^, COO^−^, and NR_4_^+^ groups [11,165]. The main structures that show the interactions between PSs and nanosurfaces are shown in Figure 4 [106,166]. When PSs are loaded into nanocarriers, micelles, liposomes, and nanospheres, PSs interact with carbon nanotubes, graphene, and nanoparticles through conjugation and chemical interactions.

Many applications combining natural PSs with nanotechnology have been developed, and these studies are summarized in Table 4. For instance, curcumin is an improved nanotechnology using many different polymers, such as polylactic acid (PLA) and poly(D,L-lactide-co-glycolide) (PLGA) [55,167], and these polymers improve the hemocompatibility and solubility of curcumin. Liposomes and serum albumin nanocarriers are another example of nanotechnology-based PS applications, and in those studies, hypericin improved the APDT performance and speed [168,169]. Graphene interactions with chlorin PS improved antibacterial efficiency [170], and hypocrellin A micelles exhibit improved solubility and overcome the solubility problem of APDT [171].

### 8.5. Computational Simulations of APDT

In addition to preclinical in vivo experiments and clinical trials, some APDT studies have focused on computational simulations. Simulations can optimize new APDT methods by optimizing treatment conditions for real human skin. For instance, Walter et al. [97] used Monte Carlo simulation to predict APDT in human skin using a porphyrin-derivative PS. Simulations provide APDT conditions, including the wavelength of light, with the highest efficiency.

### 8.6. Sonodynamic Therapy (SDT) for Enhanced Efficiency

Some molecules are activated using ultrasound energy between 1 and 2 MHz and a density of 0.5 and 10 W/cm^2^ [23]. Ultrasound enhances drug transport across the cell membrane and penetrates deeper in tissues than light [23]. Moreover, SDT with ultrasound kills bacteria, as described in the study by Costley [177]. In that study, rose bengal PS was an effective sonodynamic method that killed *S. aureus* and *P. aeruginosa*. In addition to SDT itself, SDT can be applied in combination with APDT. For instance, Alves et al. [178] reported that APDT/SDT with a chlorin derivative PS resulted in thinner biofilms and increased *C. albicans* death. Additionally, APDT/SDT treatment showed a greater antibacterial effect on *S. aureus* using curcumin as a PS [179].

## 9. Conclusions and Future Perspectives

Compared to traditional antibacterial drugs, APDT eliminates bacteria and other pathogenic organisms by overcoming drug resistance. In APDT, molecular oxygen combines with a natural PS and light of the appropriate wavelength to form cytotoxic ROS. APDT is effective against a broad spectrum of pathogenic microorganisms that cause many diseases and exhibits better tissue specificity than conventional antibiotic therapy. Various protocols have been developed using different PSs and illumination devices to minimize and eliminate obstacles that may be encountered in clinical practice. Various experimental systems, such as in vitro tests, in vivo preclinical animal models, and computational simulations, will ultimately facilitate the development of new APDT methods that are safe and effective in human clinical trials. Currently, many PS studies are examining natural extracts and compounds, their synthetic derivatives, and nanoprocessing. New natural compounds and extract PSs should be discovered and new PS structures should be developed to determine the most suitable PS for clinical applications. In addition, the use of emerging technologies such as cell-surface engineering, protein engineering, and nanotechnology to improve the efficiency and selectivity of current APDT will contribute to effectively curing human infectious diseases with no drug resistance and few side effects.

## Figures and Tables

**Table 1:** Natural compound PSs used for APDT.

Chemical Class	Name	Wavelength (nm)	Concentrations of PS	Exposure Time (min)	Light Intensity (W/m^2^)	Target Organisms	Aim	Reference
Curcuminoids	Curcumin	430	814 µM	1–5	107	*L. monocytogenes*, *Salmonella* sp.	Poultry	[78]
		455–660	0–5 µM	0–60	18	*L. monocytogenes*	Food-related diseases	[79]
		450–470	5–40 µM	0.34–1	12	*P. gingivalis, A. actinomycetemcomitans*	Periodontitis	[82]
		455–460	1.0 µM	5–60	38	*V. parahaemolyticus*	Seafood pathogens	[80]
		450	6.1 mM	4	400	*S. pyogenes*	Pharyngotonsillitis	[84]
		420	300 µM	5–25	0.3–0.6	*E. coli*	Fresh-cut food	[87]
		470	30 µM	1.5	1327	*Pseudomonas* sp.	Inactivation of spoilage organism	[81]
		420	0.5–50 µM	5–30	2980	*E. coli*	Storage quality of fresh-cut apples	[86]
		405	6.75 mM	0.22–044	3846	*P. aeruginosa*	Biofilm inhibition	[35]
		450	1.5%	12.5	800	*Staphylococcus* sp.	Rat model of wound healing	[83]
		Blue light	25–200 µM	30	30	*A. baumannii*	Multidrug resistance	[77]
		450	100 µg/mL	3.4–8.4	1500	*S. aureus*	Multidrug resistance	[88]
		470	0.5 µM	N.A.	N.A.	*E. coli*	Shelf life of fruits	[90]
		450	4.1 mM	5	670	*E. faecalis,* *C. albicans*	Endodontic treatment	[89]
		460	10–50%	120	220	Mosquito larvae	Mosquito larva control	[91]
Alkaloids	Pterin-6-carboxylic acid	350–750	100 µM	80	31	*K. pneumoniae*	Multidrug resistance	[85]
Perylenequinones	Hypericin	660	5–15 µg/mL	0.5–0.84	30,180	*C. acnes*	Acne vulgaris	[93]
		590	36 µM	68	164	*E. coli*	Food-borne pathogens	[94]
		590	10 µM	60	150	*P. aeruginosa*	Pathogen control in a worm model	[36]
	Hypocrellin	400–780	1.0 µg/mL	30	1590	*C. albicans*	Antifungal effect	[95]
Anthraquinones	Parietin	420	0.125–250 µg/mL	30	1000	*S. aureus*, *S. epidermidis*, *E. coli, P. aeruginosa*	Local infections	[96]
	Anthraquinone	410	2.5–40 µM	12	37	*L. amazonensis*	Cutaneous leishmaniasis	[50]
	Aloe emodin	460	300–500 µM	7.2	0.3–0.6	*S. aureus, E. coli*	Food-related diseases	[97]
		435	0.5–100 µM	10–40	800	*A. baumannii*	Multidrug resistant *A. baumannii*	[98]
		400–780	5–100 µM	20	800	*C. albicans*	Drug-resistant *C. albicans*	[99]
Flavins	Riboflavin	460	100 µM	90	150–580	*L. monocytogenes*	Listeriosis	[100]
Chlorin-type compounds	Chlorophyllin sodium salt	405	15 mM	10–50	96	*F. oxysporum, E. coli, T. aestivum*	Microbial control of contaminated wheat sprouts and seeds	[101]
		395	1–100 µM	30	148	*R. fascians, E. amylovora, X. axonopodis*	Plant pathogens and diseases	[102]
Porphyrin precursor	5-aminolevulinic acid	635	50–300 mM	10–30	600	*C. parapsilosis*	Trichosporonosis	[92]

N.A., Not available.

**Table 2:** Natural extract PSs used for APDT.

Extract Name	Wavelength (nm)	Concentration of PS	Exposure Time (min)	Light Intensity (W/m^2^)	Target Organisms	Aim	Reference
*B. orellana* extract	395−480	20% *w*/*v*	0.34	1530	Clinical trial	Halitosis treatment	[111]
		20% spray	0.34	1530	Biofilms of Gram-negative bacteria	Halitosis treatment	[122]
*C. longa* extract	420–480	0.78 µg/mL	1	2800	*A. actinomycetemcomitans*	Aggressive periodontitis	[113]
		25 µg/mg gel	2	12,000	*F. nucleatum, P. intermedia*	Adjunct treatment	[114]
*C. xanthorrhiza* extract	405	10–10^4^ ng/mL	5	845	*S. mutans*	Dental caries	[115]
*G. blepharophylla* extract	660	50% *v*/*v*	5	920	*S. aureus*, *S. epidermidis*, *E. coli*, *C. albicans*, *C. dubliniensis*	Antibacterial and antifungal effect	[123]
*H. sabdariffa* extract	420	0.0625–1 mg/mL	2–20	100	*E. coli, B. subtilis*	Photodynamic sanitation of foods	[119]
*I. truxillensis* extract	660	12.5 mg/mL	5	920	*S. aureus*, *S. epidermidis*, *E. coli, P. vulgaris*	Multidrug-resistant microbial infections	[110]
Mixed essential oils	660	N.A.	1	400	Natural saliva *S. aureus, E. coli*	Oral cavity microbial films	[112]
*P. obscurum* extract	315–400	0.98–1.95 µg/mL	60	34	25 clinical strains of *Candida* sp.	Oropharyngeal candidiasis	[120]
*T. minuta* extract	315–400	50 µg/mL	60	34	*C. albicans, C. krusei, C. parapsilosis, C. tropicalis, C. glabrata, T. rubrum*, *T. interdigitale*	Inhibition of virulence factors for *Candida* sp.	[118]
*E. microcorys* leaf extract	tungsten filament lamps	0.05–2%	0–1440	15	*E. coli*	Bacterial inhibition	[121]
*H. perforatum* extract (St. John’s wort extract)	570–1400	32 mg/mL	5	2000	31 different cultivable species	Oral biofilms	[116]
	sunlight	5 g/L	90	1000	Bacteriophage MS2	Human enteric virus	[117]
*T. wilfordii* extract and fraction	660	20 µg/mL	10–30	120,600	*S. aureus*, MRSA, *S. epidermidis*, *S. pyogenes*, *C. albicans*	Skin pathogens	[56]

N.A., Not available.

**Table 3:** Synthetic derivative PSs for APDT.

Chemical Class	Abbreviation	Wavelength (nm)	Concentration of PS	Exposure Time (min)	Light Intensity (W/m^2^)	Target Organisms	Aim	Reference
Perylenequinone derivatives	Hy-g	590	0.2 mg/mL	6	950	N.A.	Periodontal disease	[132]
Phenalenone derivatives	PNPPh^3 +^	463	0.5–50 µM	3.63–12	167	*S. aureus*, *E. faecalis*, *E. coli*	Selective killing	[133]
Triphenylphosphonium derivatives	Perylene derivatives	463	0.5–50 µM	3.63–12	167	*S. aureus*, *E. faecalis*, *E. coli*	Selective killing	[133]
Porphyrin derivatives	4-PtTPyP	400–800	0.91 µM	15/30	1000/500	Bovine viruses	Pharmaceutical contamination	[134]
	H_2_TMeP, ZnTMeP	380–700	5 µM	0–90	250	MRSA	Canine otitis	[108]
	TMePyP^+^	370–800	50 µM	90	500	*M. massiliense, M. fortuitum*	Mycobacteriosis	[135]
	P_M,_ P_E_, P_PN_, P_PL_	655	12.50 µM	15	1667	MRSA	Wound infections	[136]
Phthalocyanine derivatives	Es-SiPc	390–700	32–64 µg/mL	10–40	125	*S. aureus*, *E. coli*	Infectious diseases	[137]
	RLP068/Cl	600–700	1.2–36 µg/mL	10	500	*P. aeruginosa, S. aureus, C. albicans*	Localized infections	[142]
	ZnPc	390–700	64 µg/mL	120	125	*C. albicans*	HIV-infected patients susceptible to fungal infections	[138]
	ZnPcChol_8_	680	250 µM	60	278	32 clinical *P. aeruginosa* isolates	Infectious lesions	[139]
Chlorin derivatives	Zn^2+^-chlorin, Zn^2+^-mesochlorin	425	100 nM	30	56	MRSA, *E. coli*, *C. albicans*	APDT efficiency is related to the cationic charges of PSs	[141]
Bacteriochlorin derivatives	(3-PyEPy) 4BCBr8	680	250 µM	60	278	32 clinical *P. aeruginosa* isolate	Infectious lesions	[139]

Hy-g, hypericin-glucamine; PNPPh^3+^, (2-((1-oxo-1H-phenalen-2-yl)methoxy) ethyl) triphenylphosphonium bromide; 4-PtTPyP, meso-tetra(4-pyridyl)porphyrins; H_2_TMeP, free-base porphyrin; ZnTMeP, zinc (II) derivative porphyrin; TMePyP^+^, meso-tetra(N-methyl-4-pyridyl) porphyrin tetrachloride salt; P_M_, phthalocyanine with a –CH_3_ group; P_E_, phthalocyanine with a –C_2_H_5_ group; P_PN_, phthalocyanine with a –C_3_H_7_ group; P_PL_, phthalocyanine with a –C_4_H_7_O group; Es-SiPc, bis({4-[(1E)-3-oxo-3-(2-thienyl)prop-1-en-1-yl]phen-oxy})phthalocyaninato silicon(IV); RLP068/Cl, tetracationic Zn(II) phthalocyanine chloride; ZnPc, Zn(II) phthalocyanine; ZnPcChol_8_, zinc octakis(cholinyl)phthalocyanine; (3-PyEPy)4BCBr8: octacationic bacteriochlorin derivative; N.A., Not available.

**Table 4:** Natural PSs combined with nanotechnology.

Natural PS	Nanostructure	Wavelength (nm)	Concentration of PS	Exposure Time (min)	Light Intensity (W/m^2^)	Target Organisms	Aim	Property	Reference
Curcumin	PLGA NPs	447	0.2 mg/mL	0–180	1000	*S. saprophyticus*	Bloodstream infections	Hemocompatible APDT treatment	[167]
	PLA dextran NPs	455	260 µM	1–20	360	*C. albicans*	Oral candidiasis	Improved water solubility of curcumin	[55]
	Silica NPs	465	50, 1000 µg/mL	10	334	*P. aeruginosa, S. aureus*	Wound healing	Decreased hydrophobicity	[172]
	Carbogel	430	20 µM	5	12,000	*E. faecalis*	Root canals	Decreased the bacterial inactivation	[173]
	N-CUR@ICG-Met	450, 810	10 µL	5	2000	*E. faecalis*	Adjunct endodontic treatment	Improved antibiofilm activity	[174]
Hypericin	Liposomes	589	0.005–0.01 µg/µL	6	617	*S. saprophyticus*	Liposomes as carriers	Improved APDT	[168]
	Hyp-HPβCD-inclusion complex	589	8 µg	12	127	*S. saprophyticus*	Catheter surfaces	Deeper penetration into the biofilm	[175]
	Serum albumin nanocarriers	515	10 µM	30	164	*S. aureus*	Food packaging	Quick delivery to bacteria	[169]
Hypocrellin A	mPEG-PCL micelles	470	250–500 mg/L	60	900	MRSA	MRSA infections	Effective without any water solubility problem	[171]
*Tetragonia tetragonoides* extract	Nanocarrier micelle structures	632	0.06–7.9 mg/mL	20	34	*S. aureus*	Drug resistance in pneumonia	Increased biocompatibility	[74]
Chlorin e6	Graphene	650	5 mg/mL	30	22	*S. aureus*	Drug-resistant bacteria	Improved antibacterial efficiency	[170]
Pterin	Silicon surfaces	365	N.A.	20	63.7	*S. aureus*	Microbial biofilms	84.3% reduction in viable cells	[176]

PLGA, poly(D,L-lactide-co-glycolide); NP, nanoparticles; PLA, polylactic acid; N-CUR@ICG-Met, nanocurcumin with indocyanine green and metformin; Hyp-HPβCD, (2-hydroxypropyl)-beta-cyclodextrin; mPEG-PCL, methoxy poly(ethylene glycol)-block-poly(ε-caprolactone); N.A., Not available.

## Data Availability

Not applicable.
